# A Genome-Wide Characterization of MicroRNA Genes in Maize

**DOI:** 10.1371/journal.pgen.1000716

**Published:** 2009-11-20

**Authors:** Lifang Zhang, Jer-Ming Chia, Sunita Kumari, Joshua C. Stein, Zhijie Liu, Apurva Narechania, Christopher A. Maher, Katherine Guill, Michael D. McMullen, Doreen Ware

**Affiliations:** 1 Cold Spring Harbor Laboratory, Cold Spring Harbor, New York, United States of America; 2 Plant Genetics Research Unit, United States Department of Agriculture–Agriculture Research Service, Columbia, Missouri, United States of America; 3 Division of Plant Sciences, University of Missouri Columbia, Columbia, Missouri, United States of America; 4 Plant, Soil, and Nutrition Research Unit, United States Department of Agriculture–Agriculture Research Service, Ithaca, New York, United States of America; The Salk Institute for Biological Studies, United States of America

## Abstract

MicroRNAs (miRNAs) are small, non-coding RNAs that play essential roles in plant growth, development, and stress response. We conducted a genome-wide survey of maize miRNA genes, characterizing their structure, expression, and evolution. Computational approaches based on homology and secondary structure modeling identified 150 high-confidence genes within 26 miRNA families. For 25 families, expression was verified by deep-sequencing of small RNA libraries that were prepared from an assortment of maize tissues. PCR–RACE amplification of 68 miRNA transcript precursors, representing 18 families conserved across several plant species, showed that splice variation and the use of alternative transcriptional start and stop sites is common within this class of genes. Comparison of sequence variation data from diverse maize inbred lines versus teosinte accessions suggest that the mature miRNAs are under strong purifying selection while the flanking sequences evolve equivalently to other genes. Since maize is derived from an ancient tetraploid, the effect of whole-genome duplication on miRNA evolution was examined. We found that, like protein-coding genes, duplicated miRNA genes underwent extensive gene-loss, with ∼35% of ancestral sites retained as duplicate homoeologous miRNA genes. This number is higher than that observed with protein-coding genes. A search for putative miRNA targets indicated bias towards genes in regulatory and metabolic pathways. As maize is one of the principal models for plant growth and development, this study will serve as a foundation for future research into the functional roles of miRNA genes.

## Author Summary

MicroRNAs are non-coding RNAs that regulate gene expression post-transcriptionally and play roles in diverse pathways including those acting on development and responses to stress. Here, we describe a genome-wide computational prediction of maize miRNA genes and their characterization with respect to expression, putative targets, evolution following whole genome duplication, and allelic diversity. The structures of unprocessed primary miRNA transcripts were determined by 5′ RACE and 3′ RACE. Expression profiles were surveyed in five tissue types by deep-sequencing of small RNA libraries. We predicted miRNA targets computationally based on the most recent maize protein annotations. Analysis of the predicted functions of target genes, on the basis of gene ontology, supported their roles in regulatory processes. We identified putative orthologs in Sorghum based on an analysis of synteny and found that maize-homoeologous miRNA genes were retained more frequently than expected. We also explored miRNA nucleotide diversity among many maize inbred lines and partially inbred teosinte lines. The results indicated that mature miRNA genes were highly conserved during their evolution. This preliminary characterization based on our findings provides a framework for future analysis of miRNA genes and their roles in key traits of maize as feed, fodder, and biofuel.

## Introduction

The last decade has witnessed remarkable progress in our knowledge of the biogenesis and activity of diverse classes of small non-coding RNAs (sRNA). These include microRNAs (miRNA) [1], small interfering RNAs (siRNA) [2], *trans*-acting siRNAs (ta-siRNA) [3], and others [4]. While the majority of plant sRNAs are transcribed from siRNA genes residing in repetitive and transposon-rich regions, and regulate chromatin silencing [4], a great deal of interest has been placed on miRNAs due to their ability to post-transcriptionally regulate gene expression [5]. This is exemplified by the critical regulatory behavior of miRNAs at key positions in a variety of pathways, such as root [6],[7], shoot [8], leaf [9]–[11] and flower [12],[13] development and cell fate [14],[15]. Additionally, they also include responses to phytohormones [16], nutrient [17]–[19] and other environmental stresses [20]–[23].

As a genetic model system, maize has contributed significantly to our understanding of plant development and evolution, and more recently this knowledge has been employed to elucidate the regulatory functions of miRNA genes. For instance, teosinte glume architecture 1 (tga1) is one of the major genes responsible for the evolution of maize from its ancestor teosinte and has also been identified as a target for miR156 [24],[25]. Intriguingly, mutations in Corngrass1 (Cg1) result in the over-expression of miR156 and decreased miR172 levels, resulting in alterations of the juvenile to adult phase transition [24],[25]. Taken together, it is apparent that miRNA regulation is intertwined with key plant development processes. Thus, there is considerable interest in taking advantage of the complete genome sequence of maize B73 reference genome version 1 (B73 RefGen_v1) [26] to systematically identify miRNA genes, their corresponding targets, and to decipher their regulatory roles.

The mature biologically active products of miRNA genes define miRNA gene families. This, along with the characteristic ‘hairpin’ structure of its precursor (pre-miRNA), allows computational detection and annotation of miRNA genes [1],[4]. Post-transcriptional regulation is accomplished by the RNA-induced silencing complex (RISC complex) which directs complementary binding of the mature miRNA product to mRNA transcripts, usually resulting in target cleavage or inhibition of translation [27]. In plants, the near perfect complementarity between the miRNA and its substrate mRNA has permitted computational methods for the specific identification of target genes [5].

In this study, we systematically annotated miRNA genes based on the first nearly complete assembly of the maize B73 RefGen_v1 [26]. This analysis includes experimental validations for many of these genes, as well as expression studies using deep small RNA transcriptome sequencing across a broad panel of maize tissues. We also conducted one of the first comprehensive characterizations of maize pri-miRNA transcripts, providing a deeper understanding of their transcription and regulation. Prediction of protein-coding targets confirmed many known regulatory substrates and provided a window into many more potentially novel pathways. We also describe the first analysis of allelic diversity of miRNAs in maize using a large panel of highly diverse inbred lines as well as its wild relative teosinte. A comparative genomic analysis with sorghum provided insight into the evolutionary dynamics of miRNA family expansions and will serve as basis for future comparative functional genomic analyses using syntenic orthologs. We believe the multi-faceted nature of this study will help accelerate our understanding of miRNAs, their regulatory roles in critical biological processes, as well as offer the community detailed annotations applicable to their own research.

## Results

### Genome-wide survey of known pre–miRNAs and their paralogous pre–miRNA structures in maize

Starting with a set of 1822 plant mature miRNA sequences available in miRBase [28], we predicted putative pre-miRNA structures at more than 4,000 loci in the maize B73 RefGen_v1 [26]. Of these, more than 300 passed initial filters for positional overlaps, secondary structure and orientation of mature miRNA sequences within the respective stem-loop structures. After additional screening for overlaps with transposable elements (Table S1) and removal of families not well characterized in current literature, we annotated 150 miRNAs from 26 families with high confidence. Since our search space for pre-miRNA structure included only 250nt surrounding the aligned mature miRNA, our method may have missed families that have introns in their precursors. One example is miR444, which has been reported as conserved in rice, maize, sorghum, and sugarcane [29]–[31], but was missed by our pipeline due to a large intron in its pre-miRNA structure.

There are a total of 98 maize miRNA genes deposited in miRBase [32]–[34], which were predicted based on the MAGI (Maize Assembled Genomic Islands: maizegdb.org) and TIGR EST collections. Of these, 89 were detected in our set and are now annotated on the B73 reference genome assembly. We failed to detect nine miRNA genes in our pipeline: miR160e, miR166e, miR166f, miR169e, miR169g, miR169h, miR172a, miR172e and miR408. It is possible that these are located in regions that are not captured in the current genome assembly. In addition to those previously characterized, we found 61 putative miRNA genes representing new members of conserved families. A summary of known and previously identified miRNA gene families is provided in Table 1. Detailed information about the genomic location of each miRNA, as well as the mature miRNA and miRNA* for each miRNA gene, is listed in Table S2.

**Table 1:** Maize miRNA gene summary.

Family	No. of Known miRNA Genes	No. of Paralogous miRNA Genes	Total
miR156	11	1	12
miR159	4	7	11
miR160	5	2	7
miR162	1	0	1
miR164	4	4	8
miR166	11	1	12
miR167	9	1	10
miR168	2	0	2
miR169	8	7	15
miR171	11	3	14
miR172	3	0	3
miR319	4	0	4
miR390	0	2	2
miR393	1	2	3
miR394	2	0	2
miR395	3	13	16
miR396	4	3	7
miR397	0	2	2
miR398	0	2	2
miR399	6	4	10
miR408	0	1	1
miR482	0	1	1
miR528	0	2	2
miR529	0	1	1
miR827	0	1	1
miR1432	0	1	1
Total	89	61	150

Breakdown of miRNA genes identified in this study. Known miRNA genes are ones previously deposited in miRBase while paralogous miRNA genes are previously uncharacterized members of conserved miRNA families.

Annotated miRNA genes were found on all maize chromosomes, having a distribution pattern similar to those of protein-coding genes (Table S2) [26]. No miRNA genes were found in unanchored portions of the assembly. Like protein coding genes, a proportion of the miRNA genes are organized as tandem paralog clusters (see section “Genome Organization and Conservation with Sorghum”). However, a subset of these was found as unusually compact clusters, with less than 2000nt separating adjacent genes, as shown in Table 2. Two members of the miRNA156 family (miR156b and miR156c) on chromosome 3 are separated by less than 200nt, as are two members of the miRNA166 family on chromosome 5. The close distance of miR156b and miR156c is observed in several monocots and they are transcribed as one transcript (polycistronic) in maize [24] and rice [35]. Clusters of two members of miR166 are also observed in several plant species, including moss [33]–[37]. Compact clustering was particularly prevalent in the miR395 family. Four such clusters were found on chromosomes 2 and 10, comprising 16 genes in total. In rice, compact clusters of miR395 genes have also been observed with each forming a single polycistronic transcription unit [38].

**Table 2:** Maize miRNA gene clusters.

Family	miRNA Gene	Chromosome	Start	End	Strand	Distance^a^
miR156	miR156c	chr3	7601812	7601915	+	
	miR156b		7602113	7602200	+	198
miR166	miR166k	chr5	209830443	209830572	−	
	miR166m		209830714	209830831	−	142
miR167	miR167a	chr3	115392919	115393077	+	
	miR167g		115394886	115395094	+	1809
miR395	miR395d	chr2	6308283	6308349	−	
	miR395e		6307947	6308031	−	414
	miR395f		6308123	6308193	−	92
	miR395g		6307467	6307533	−	90
miR395	miR395h	chr2	6317588	6317673	−	
	miR395i		6317765	6317835	−	92
	miR395j		6318406	6318491	−	571
	miR395b		6318583	6318658	−	92
	miR395a		6319238	6319348	−	580
miR395	miR395k	chr10	78978258	78978342	−	
	miR395l		78978429	78978510	−	87
	miR395m		78978558	78978640	−	48
	miR395c		78978705	78978842	−	65
miR395	miR395n	chr10	144308658	144308724	+	
	miR395o		144308831	144309057	+	107
	miR395p		144309149	144309238	+	92

a distance (nt) to previous miRNA gene in the cluster.

MicroRNA gene families existing as clusters are listed in this table. Sixteen miR395 genes are organized into 4 compact clusters on two chromosomes, with the first two clusters 10 kb apart. RACE data from this study confirms that the miR156c/miR156b cluster and the miR166k/miR166m cluster are polycistronic.

### Characterization of the pri–miRNA

Primary transcripts of miRNA genes are known to have features typical of transcription by RNA polymerase II, including 5′ capping, 3′ polyadenylation, and intron splicing [39]. To characterize the pri-miRNA transcripts in maize, we designed gene-specific primers for each of the 89 previously identified miRNA genes and conducted 5′ RACE and 3′ RACE using a template containing mixed tissues of seedling, immature tassel and immature ear. Overall, we were able to capture the upstream transcribed regions (5′ region) of 55 miRNA genes and the downstream transcribed regions (3′ region) of 51 miRNA genes (Table S2). Among these, we obtained both 5′ and 3′ RACE products for 40 miRNA genes, producing full-length transcript sequences. Failure to amplify some miRNA gene products might have been a limitation of the tissues sampled, as some miRNA genes might be expressed in highly specific tissue/cell types, developmental stages, or environmental conditions. From our RACE data, we were able to confirm the clusters of miR156b/c and miR166k/m, but failed to detect miR395a/b clusters. Genes of miR395 family are known to be up-regulated in response to low-sulfate conditions [40] and it is possible that transcript abundance was below the detection threshold given the normal nutrient status in which our plants were grown. While our work was underway, the data for maize full-length complementary DNA (FLcDNA) became available [41],[42]. To identify pri-miRNAs in this FLcDNA set, we mapped 150 miRNA precursors against 63,000 FLcDNAs and found 33 transcripts that harbor 27 pre-miRNA sequences. After excluding those pri-miRNAs that were mapped with RACE, we identified 10 additional pri-miRNAs using FLcDNA analysis (Table S3).

**Figure 1:**
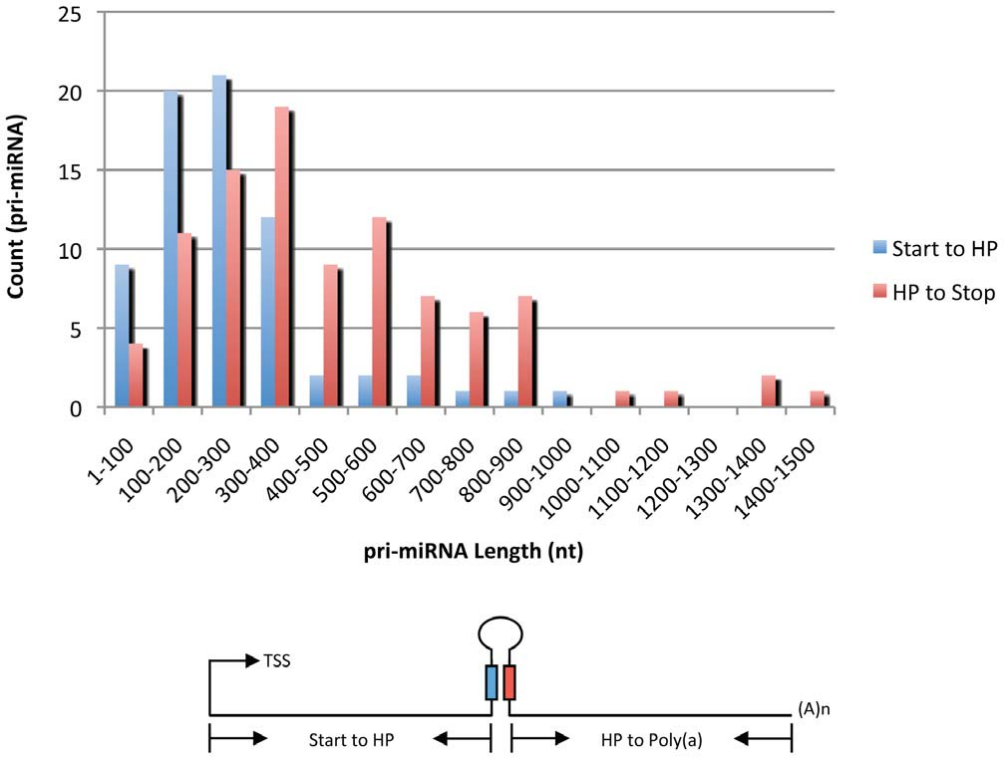
Length distribution of pri-miRNA. Length distribution of 5′ (blue bars) and 3′ (red bars) regions of pri-miRNA. The 5′ region is defined as the segment from the transcription start site to the hairpin structure (HP), while the 3′ region is the segment between the hairpin structure and the transcription stop site, 3′ regions are longer, averaging 953nt versus 523nt.

**Figure 2:**
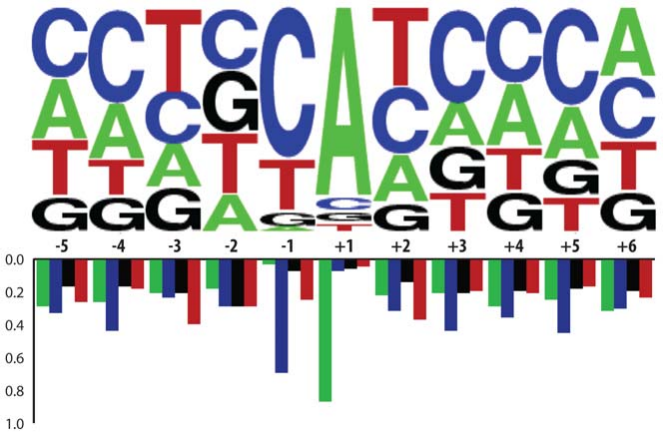
Nucleotide frequency surrounding pri–miRNA transcriptional start sites. Nucleotide frequencies surrounding transcriptional start sites (as determined from 5′ RACE products) are shown using sequence logos [121],[122]. TSSs are predominately marked by an adenine at the +1 position and a cytosine at the −1 position.

**Figure 3:**
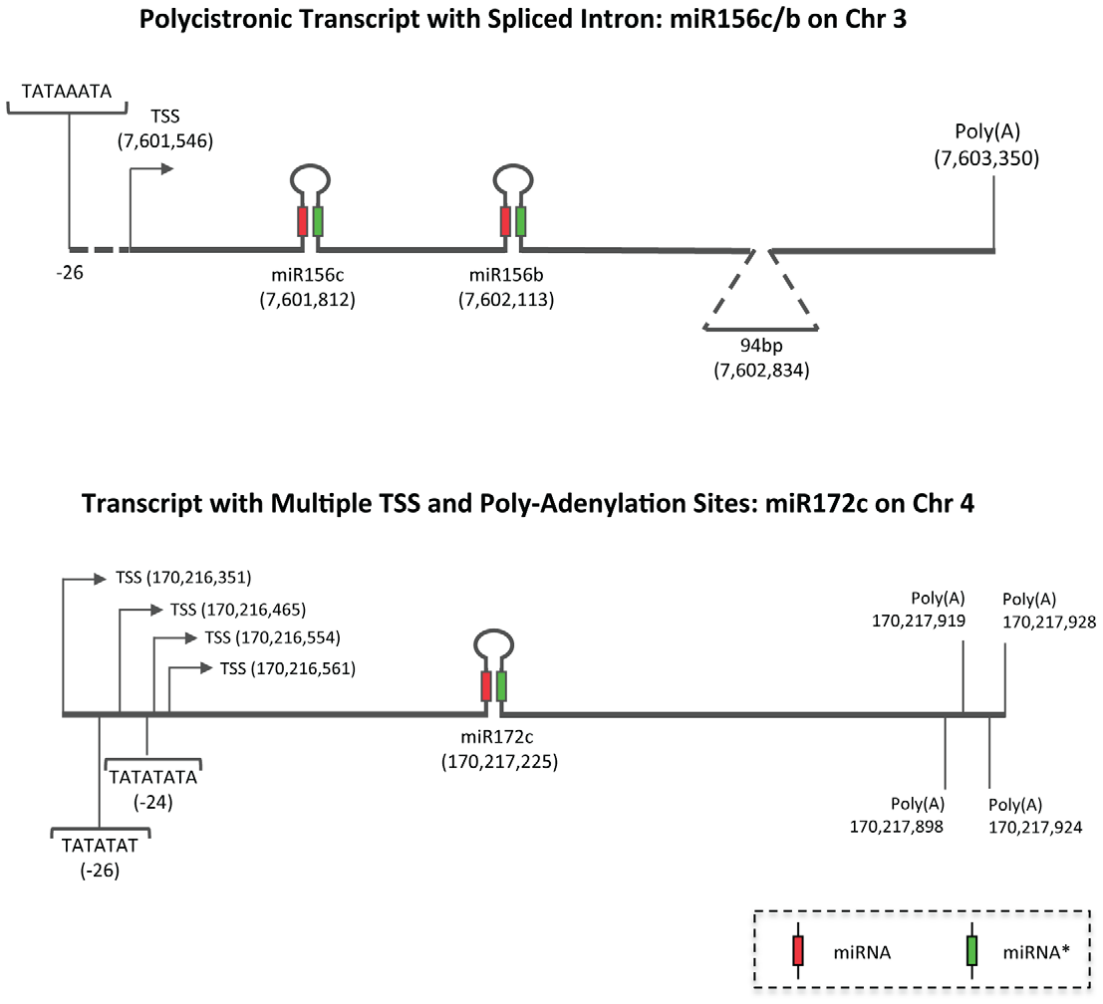
Schematic representation of splicing variants for maize miRNA primary transcripts. Examples of introns, alternative transcriptional start sites (TSSs), polyadenylation sites, and polycistronic features of pri-miRNAs seen in miRNA RACE amplicons mapped to the maize genome. Mature miRNAs are represented by red boxes, miRNA* by green boxes. miRNA precursors are represented by hairpin structures while TATA box-like motifs are also indicated in the figure. Numbers in parentheses indicate physical locations on the reference chromosome. (A) RACE products show that miRNAs pri-miR156b and pri-miR156c are transcribed as a single transcript, with a 94nt intron spliced out from the locus. (B) pri-miR172c is an example of a miRNA having multiple TSSs and polyadenylation sites. 5′ RACE products map to 4 distinct locations and 3′ RACE products carry 4 distinct polyadenylation signatures. Two of the TSSs are associated with TATA box-like motifs.

The overall lengths for pri-miRNA transcripts ranged from 250nt to nearly 2000nt, with an average size of 810nt, far less than the 1,433nt average for maize protein-coding transcripts [26]. Figure 1 shows that the 3′ region of pri-miRNAs (measured from the stem-loop to the transcriptional stop site) is generally longer than the 5′ region (measured from the transcriptional start site (TSS) to the stem-loop), having mean lengths of 953nt and 523nt respectively. We observed that 83% of maize pri-miRNAs have TATA-box like motifs in the −23 to −28 positions relative to TSS (data not shown). Previous studies using rice and Arabidopsis FLcDNAs and Arabidopsis pri-miRNAs showed a high prevalence of an adenine at the TSS and a cytosine at the −1 position [43]. Of the available 76 miRNA gene transcripts with TSS sites, most have A at the TSS and C at the −1 position (Figure 2). We also observed evidence for alternative TSS. Among the 55 miRNA transcripts with 5′ region information, 10 had two TSSs that are 3nt to 9nt apart. This is similar to FLcDNAs studies of Arabidopsis and rice showing two TSSs per locus, with an average distance of 4.2nt and 8.7nt apart, respectively [44]; however, the distance between various stop sites can be up to several hundred nucleotides. Examples of polycistronic transcripts and multiple TSS and stop sites are shown in Figure 3.

In addition we found that introns were more abundant in the 3′ region than in the 5′ region. From RACE reaction products, we found only one gene (miR164c) with an intron in the 5′ region, whereas 15 genes showed one or two introns in the 3′ region (Table S4). Intron length varied from 71nt to 2196nt, and the canonical splice motif GU…AG was found for all but one intron (Table S4). Alternative non-spliced transcripts were found for three genes: miR159a, miR166g, and miR169i.

### Expression profiles of miRNAs

Using Illumina's sequencing-by-synthesis technology [45], we profiled the genome-wide transcript profiles of miRNA abundance across five different maize tissue types (root, seedling, tassel, ear, and pollen). Small RNA libraries were generated for each tissue and sequenced using the Illumina 1G Sequencer. The small RNAs (18–22nt) were mapped to the predicted pre-miRNAs. The number of reads mapping to each pre-miRNA were enumerated and normalized against the total count of 18 to 22 nucleotide reads, reported as reads per million (RPM), for each respective library. A summary of the quantitative expression profiles for each of the 26 miRNA families is shown in Table 3. Of the five tissues surveyed, the seedling samples showed the highest expression levels while the pollen samples showed the lowest expression levels, with RPM counts an order of magnitude larger in the seedling. We noted that miR482 lacks an expression signature in any of the five tissues, while miR162 miR394, miR395, miR398, miR399, miR408, miR528 and miR1432 had low expression counts (less than 200 RPM). In contrast, miR156, miR159, miR167, miR168, miR169, miR171, miR319, and miR529 had high expression counts (slightly over 3,000 RPM, on average). In our samples, miR529 was over-expressed in the tassel (almost 16,000 RPM) but under-expressed in the other four tissues (slightly over 300RPM, on average). Several families showed higher expression levels in juvenile tissues. The miR156, miR164, miR168, miR393, miR395, miR396, miR398, and miR399 families had higher signatures in juvenile root and seedling tissues while miR172 demonstrated a higher expression level in reproductive tissues (tassel and ear).

**Table 3:** Expression levels of maize miRNA genes assessed using Solexa sequencing.

Family	Tissue				
	Root	Seedling	Tassel	Ear	Pollen
miR156	6984.9	14958.61	206.96	382.82	818.55
miR159	6746.77	14015.49	5522.09	4052.65	1058.72
miR160	362.44	299.51	130.31	256.59	11.87
miR162	1.12	2.74	8.4	13.2	0.61
miR164	1408.21	2168.61	428.51	488.43	26.79
miR166	1140.5	503.64	600.06	518.96	127.55
miR167	8602.42	25826.68	8040.59	7664.73	428.3
miR168	15399.73	10368.55	2472.15	3877.74	4046.77
miR169	5973.95	16692.19	975.28	1118.77	64.53
miR171	4035.93	15764.5	8050.45	5335.61	869.08
miR172	108.58	162.28	885.49	632.81	21
miR319	8378.89	3420.85	2625.81	14443.36	184.47
miR390	280.82	2220.41	1059.23	339.1	170.47
miR393	500.98	644.99	153.67	74.25	16.74
miR394	6.37	4.46	38.69	9.08	0.61
miR395	36.32	41.51	12.05	0	0
miR396	1790.5	3068.5	83.22	57.75	504.71
miR397	250.86	556.13	128.12	16.5	169.25
miR398	67.77	118.36	38.33	0	16.13
miR399	144.53	249.08	15.7	39.6	4.57
miR408	74.14	39.45	15.33	0	11.87
miR482	0	0	0	0	0
miR528	128.8	110.13	309.16	11.55	20.09
miR529	644.01	359.2	15899.05	86.63	169.25
miR827	604.32	711.89	395.66	364.67	31.66
miR1432	4.49	16.12	0	0	0.61
Total	63659.76	112298.83	47543.89	39336.8	8768.72

Small RNA libraries were constructed for a variety of maize tissues and sequenced using Illumina's sequencing-by-synthesis technology [45]. The level of expression of miRNA gene families in each tissue was assessed by counting the number of 18–22 nt reads mapping to each family, normalized by the total number of 18–22 nt reads in the respective libraries. Counts are in Reads per Million (RPM).

**Figure 4:**
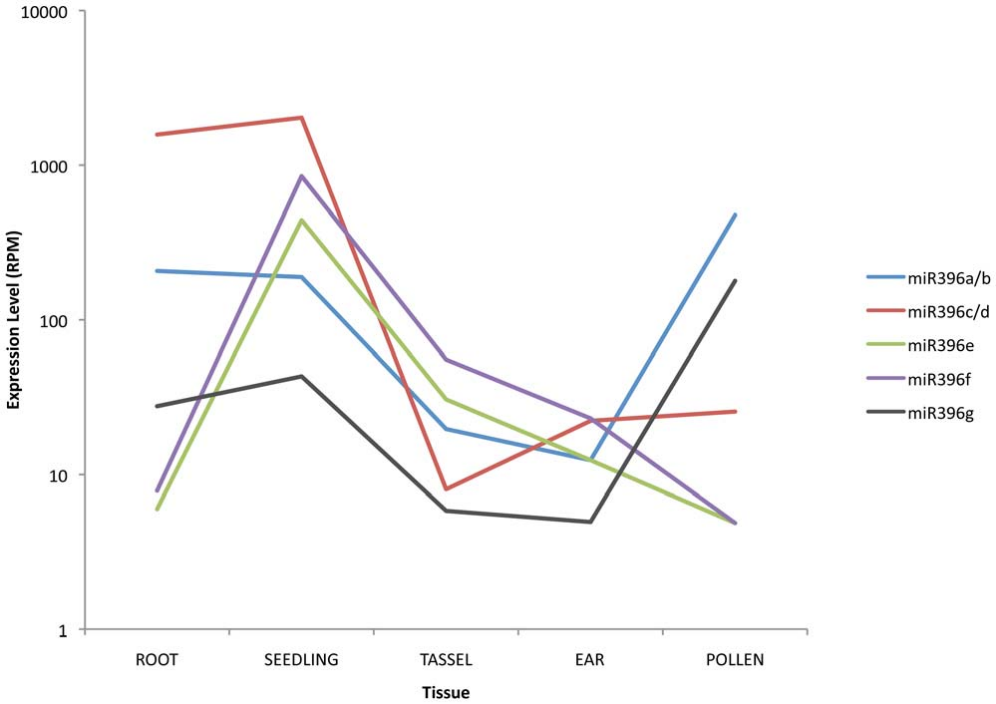
Expression levels of miRNA genes from miR396 family across tissue types. Solexa reads mapping to the miR396 family were counted and summed for each family member, showing tissue specificity of expression amongst members of the same family. Read counts are plotted as Reads Per Million (RPM) on a log scale.

Within miRNA families, there are examples of tissue-specific expression differences among individual miRNAs. For example, the miR396 family is overall highly expressed in the juvenile root and seedling samples but only certain individual family members (miR396a, miR396b, and miR396g) are highly expressed in pollen (Figure 4).

### Prediction of miRNA protein-coding targets

We computationally predicted potential maize targets using the Filtered Gene Set transcripts (Release 4a.53) of the B73 maize genome sequence [26]. The filtered set of transcripts contains about 32,500 entries, and is comprised of the longest representative cDNA transcripts. These are presumed free of pseudogenes and low-complexity repetitive elements such as short-tandem repeats and transposons. Our computational pipeline predicted 247 unique putative genes targeted by 150 miRNA sequences belonging to 26 miRNA families. Around 85% of the predicted targets have functional InterPro annotations [46]. We observed that 12 out of 26 miRNA families are predicted to target transcription factors, as shown in Table S6, suggesting roles of these miRNA families in post-transcriptional regulation and transcription networks. In addition to transcription factors, other predicted target genes are involved in diverse physiological and metabolic processes. Such targets include protein kinases, signal transduction histidine kinases, antifreeze proteins, F-box proteins, cytochrome P450, cupredoxin, peroxidases, multicopper oxidases, transporters, ATP sulfurylases, and cell division proteins (Table S6).

### Classification of predicted miRNA target functions using Gene Ontology (GO)

**Figure 5:**
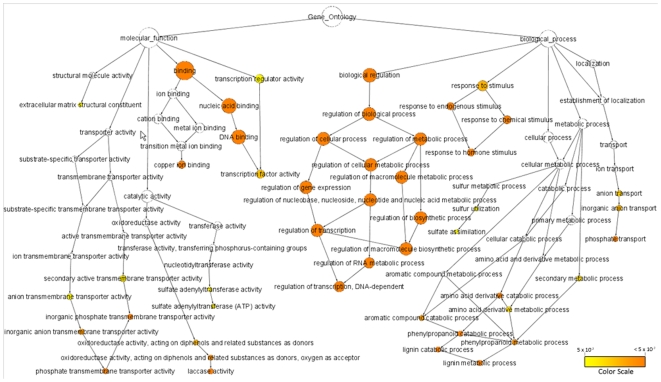
Maize miRNA targets enrichment network based on GO molecular functions and biological processes. Significantly overrepresented GO terms based on GO molecular functions and biological processes were visualized in Cytoscape. The size of a node is proportional to the number of targets in the GO category. The color represents enrichment significance— the deeper the color on a color scale, the higher the enrichment significance. White color nodes are not enriched but show the hierarchical relationship among the enriched ontology branches.

To gain a better understanding of the functional roles of the predicted miRNA target genes in maize, we looked for target enrichment in Gene Ontology (GO) molecular function and biological process categories [47]. The targets were annotated by using the GO annotations available from the B73 RefGen_v1. Of the predicted targets, 76% had GO assignments whereas only 53% of the genes in the entire refined set were associated with GO terms. BiNGO (Biological Networks Gene Ontology) [48] was used to study targets enrichment and to construct a hierarchical ontology tree in Cytoscape [49], as shown in Figure 5. We found that miRNA families preferentially target genes involved in a wide spectrum of regulatory functions and selected biological processes including gene expression/transcription, metabolism, catalysis, transport, and response to stimuli (Table S7).

The genes targeted by miRNA families showed a strong affinity for binding activity (82.3%), transcription factor activity (10.6%) and transcription regulator activity (11.2%). In the GO biological process enrichment analysis, targeted genes were found to be involved in biological regulation of cellular processes (35.2%), biosynthetic processes (32%), and metabolic processes (32%). Approximately 32% of the target genes were involved in transcription/gene expression while about 19% of targets were classified in the response to stimulus category. The latter were found to be involved in the response to abiotic stimulus, endogenous stimulus, and hormone stimulus (Table S7). Since one gene can be related to multiple GO terms, the sum of the percentages of GO terms is not a relevant parameter.

We also computationally evaluated whether specific miRNA families were preferentially enriched in certain GO categories. Target genes of the following 8 miRNA families were overrepresented in both of the biological processes i.e. the regulation of transcription and the response to stimulus. These families are: miR156, miR160, miR164, miR166, miR167, miR172, miR396, and miR528. In addition to these families, target genes of miR169, miR319, miR408 and miR529 were involved in transcription regulation, whereas miR159, miR397, and miR399 target genes were involved in response to stimulus. Targets of only miR395 family showed involvement in sulfate assimilation pathway whereas targets of both miR395 and miR399 families showed enrichment in transmembrane transport activities. Target genes of miRNA families, miR164, miR397, miR408, and miR528 showed enrichment in laccase and oxidoreductase activities and were found to be involved in secondary metabolic processes such as phenylpropanoid, amino acids, aromatic compounds and lignin catabolic processes (Table S7).

### Genome organization and conservation with sorghum

Previous studies in Arabidopsis [37],[50] and rice [38] have shown that miRNA gene families evolved from a combination of tandem, segmental, and whole-genome duplication events [51]. Maize was derived from an ancient allotetraploid, and it is estimated that the two maize progenitor genomes diverged from the ancestor of sorghum ∼12 MYA [52]. Comparative mapping between maize and sorghum can therefore reveal the fate of miRNA genes after whole genome duplication. In addition, synteny can be used to help infer orthologous relationships between these species.

**Figure 6:**
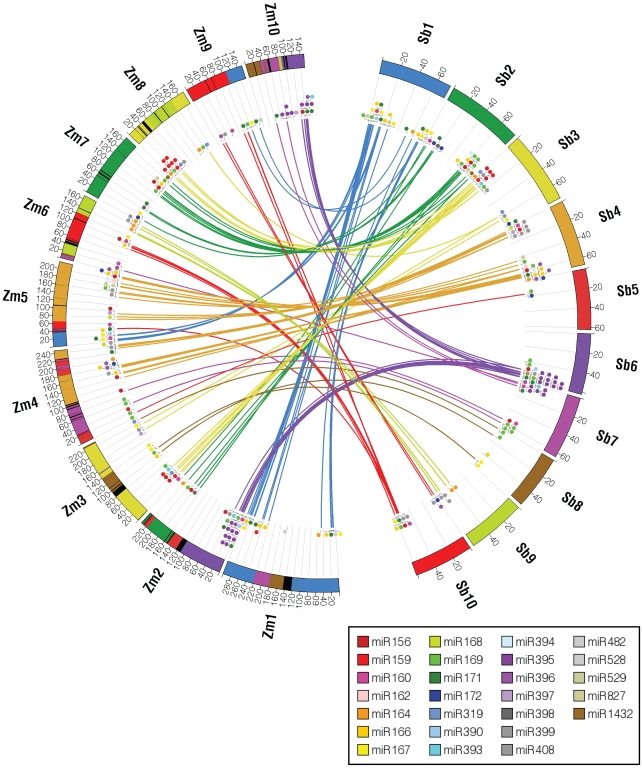
Comparative map between maize and sorghum genomes showing links between syntenic MIR genes. Positions of annotated MIR genes are shown using circles that are color-coded according to the family. Links show synteny between MIR genes. Maize and Sorghum chromosomes are color-coded such that syntenic regions, based on protein coding genes, have matching colors.

Based on the maize high confidence set, we filtered previously annotated *Sorghum bicolor* miRNA genes [29]. The distribution of these genes by family is shown in Table S8, along with corresponding information for maize. Synteny was examined in the context of orthologous protein coding genes which numbered 25,216 in maize and 20,408 in sorghum [26] (See Materials and Methods). In total, we found 136 maize and 106 sorghum miRNA genes within syntenic regions, corresponding to 91% and 79% of their respective totals. These values are similar to the percentages of syntenic protein-coding orthologs, 85% in maize and 89% in sorghum [47]. The lower percentage of syntenic sorghum miRNA genes may be indicative of false positives within this set, as these did not undergo the same rigorous screening process as for maize. Synteny was found amongst all families except miR827 and miR482 (Table S8). The former has a single representative in each genome, located in non-syntenic regions; the latter has one member in maize but none annotated in sorghum. As shown in Figure 6, conserved synteny among miRNA genes was detected on all chromosomes of maize and sorghum. This figure also shows that many miRNA genes in sorghum map to both sister sites created after the genome-wide duplication event in maize.

Many miRNA genes are organized within paralog clusters, defined as family members having no more than two intervening genes. Some of these are comprised of compact clusters, as described above. In maize, we found 13 paralog clusters containing 40 genes in total, while sorghum has 15 clusters containing 47 genes. All of the 13 paralog clusters in maize are at syntenic positions. In sorghum, 14 of the clusters (43 genes) are in syntenic regions. Since the relationship between paralog clusters is not known between species (some may be orthologs and some paralogs depending on the timing of tandem duplications relative to speciation) we collapsed these into single sites. This led to 104 sites in sorghum and 123 sites in maize. Of these, 81 sites are syntenic in sorghum, therefore representing ancestral locations of these genes in the common ancestor of maize and sorghum. In maize, 28 of these sites are retained in sister duplicate positions. Thus, almost 35% of ancestral miRNA positions have been retained at sister homoeologous positions in maize, as detailed in Table S9. This is higher than the retention rate of protein-coding genes, measured at ∼21%.

Maize had a net gain of 30 syntenic genes compared to sorghum. All of these gains occurred at retained duplicate sites. Amongst singleton sites (those harboring miRNA genes on just one sister region), there was a deficit of five maize genes due to the reduced sizes of four paralog clusters relative to sorghum. Whether this was caused by the expansion of clusters in sorghum or the contraction of clusters in maize is not known. In contrast, the combined gene counts at the 28 retained duplicate sites in maize exceed gene counts at the corresponding sorghum sites in all but two cases. This resulted in a net gain in maize of 35 genes at retained duplicate sites. There were twenty-two sites harboring single genes in sorghum with corresponding duplicate sites in maize also harboring single genes. An additional site contains exactly two genes at each corresponding region. Assuming these gene counts were stable through evolution, then a gain of 24 genes can be directly attributed to the whole genome duplication in maize. The remaining five retained sites have clusters with varying numbers of genes in sorghum and corresponding maize sister regions. These sites contributed a net gain of eleven maize genes, the combined result of whole-genome duplication and differential expansion/contraction of paralog clusters.

The evolutionary dynamics influencing miRNA family size is illustrated by closer examination of the miR159 family. This family has the largest difference in membership, 11 genes in maize compared to 3 genes in sorghum (Table S8). This excess of eight genes includes three (miR159d/e/g) that have no syntenic counterparts in sorghum, suggesting long-distance movement in maize or loss of ancestral sites in the sorghum lineage. Another four exist within a paralog cluster that is expanded in maize relative to sorghum. In maize this cluster contains six genes (miR159a/b/h/i/j/k) on chromosome 8 while the corresponding cluster on sorghum chromosome 3 has only two genes. Finally there is a single gene (miR159f) at the homoeologous site on maize chromosome 3 which resulted from the whole genome duplication.

### Nucleotide diversity of miRNA genes

Because of the characteristic structure and important regulatory function of miRNAs, it would be expected that the mature miRNAs be highly conserved across maize accessions. Sequence diversity in the flanking regions of the mature miRNA can be used to imply natural or artificial selection on the miRNA loci. To explore the genetic diversity of miRNAs in maize, we selected 28 maize miRNA genes (Table S10) based on their availability in the initial reduced representation libraries of maize [53],[54]. We designed sequencing primers for ∼400nt surrounding each mature miRNA sequence. The 28 maize miRNA genes and flanking regions were sequenced in 28 maize inbred lines chosen to maximize genetic diversity [55]. Additionally, 16 partially inbred teosinte lines [56] (*Zea mays* ssp. *parviglumis*) were selected to represent the diversity in the progenitor of cultivated maize. As expected, there was no polymorphism detected within the mature miRNA sequences in either the maize inbred or teosinte lines. However, the level of nucleotide diversity flanking the mature miRNA sequence is similar to the genic level of diversity: the average proportion of pairwise nucleotide differences per nucleotide site (π) is 0.0065 for miRNA genes in the maize inbred lines versus 0.0067 for a random collection of 1,095 maize genes[57]. By comparing genetic diversity in inbred versus teosinte accessions, we tested if any of the miRNAs had a reduction in polymorphisms consistent with the selective sweep during maize domestication or improvement. We found π = 0.0105 for the miRNA genes among the teosinte lines compared to π = 0.0095 for a large collection of teosinte protein coding genes [56]. We found no evidence of such a selective sweep (Table S11) suggesting that mature miRNA sequences are under strong purifying selection due to their functional importance, making them highly conserved over long evolutionary periods, while flanking regions evolve equivalent to other genes.

## Discussion

### Evaluation of high-confidence miRNA prediction for maize

We have systematically annotated miRNA genes on the first complete assembly of the maize genome. Our computational methods for detection were stringent by design, distinguishing 150 genes in 26 miRNA families from many potentially spurious predictions. This high confidence set represents genuine miRNA genes is reinforced by our characterization of pre-miRNA structures, promoter features, expression profiles, genetic diversity, and identification of orthologous genes in sorghum (see further discussion below). Plant miRNA families can be historically divided into two classes: the highly conserved families, and the emerging class of lineage-specific families. Recent work suggests that a subset of the predicted lineage-specific families have characteristics of siRNA genes, associated with transposon-related repeats and showing little or no expression at the pre-miRNA level [58]. One such family is miR414, which is currently under consideration for removal from miRBase [5],[28]. Another example is the miR437 family, which has expanded in the sorghum and maize lineages relative to rice. In maize, more than 90% of miR437 pre-miRNA structures show the presence of the *Stowaway* miniature inverted repeat transposonable element (MITE). Interestingly, these MITEs account for more than 87% of the repeats found in miRNA genes. In our analysis, maize transposon-related repeats were found in 11 miRNA families (miR437, miR854, miR1128, miR1132, miR1133, miR1320, miR1435, miR1436, miR1439, miR1884, and miR2102), thus making them suspect and supporting their removal from the high-confidence set (Table S1). In [27], Voinnet also noted the association of recently evolved families with MITEs and raised similar questions as to whether they represent true miRNAs versus siRNAs.

In animals, approximately 80% of miRNAs are found within introns of either protein-coding or non-coding genes [59]. In contrast, most of the annotated miRNAs in plants are located in intergenic regions, with some exceptions [60]. In the current work, 87% of the miRNAs were found in intergenic regions. Exceptions were 19 genes whose mature miRNA coordinates lie within exons of predicted protein coding genes. In six of these cases the host genes were also the predicted target of the embedded miRNA gene, oriented either on the same strand (miR159c, miR319b, miR396a and miR397a) or opposite strand (miR169j and miR399d). Of the remaining 13 miRNA genes, all except miR159e were located on the same strand as the protein-coding genes they are embedded in. miR159e was not expressed in any of the tissues sampled and has no syntenic relationship with any of the sorghum miR159 genes (see sections below), suggesting that miR159e might not be functional. The protein-coding genes with embedded miRNAs are small (encoding proteins of less than 120aa compared to the average of 358aa [26]) and have no identifiable InterPro domains. We speculate that a proportion of these may not be bona fide protein-coding genes but likely the result of misannotation based on populations of non-coding transcripts used in the evidence-based prediction method [26].

### Expression of miRNA families and genes based on small RNA library profiling

MicroRNA tissue-specificity is known to play a role in plant development, an example of which is the regulation of juvenile-to-adult vegetative phase transition [61]. To assess miRNA tissue specificity, we surveyed miRNA expression levels in five maize tissue types (root, seedling, tassel, ear, and pollen). In our data, we found that the expression of miR156 and miR172 families are anti-correlated (Table S4); miR156 is expressed higher in young roots and seedlings but lower in adult tissues (tassel, ear, and pollen), while miR172 expression has an opposite trend, albeit with a lower overall expression level. These results fit well with the current models of phase transition, whereby opposing gradients of miR156 and miR172 are responsible for the transition from juvenile to adult. This converse regulatory relationship between miR156 and miR172 has also been reported recently [62].

The miR529 family is expressed conspicuously higher in the tassel as compared with the other surveyed tissues. We note that the mature miRNA products of families miR529 and miR156 are identical from positions 8–14; which are key residues in miRNA-target recognition. Consequently both miRNA families have similar predicted targets consisting mainly of SQUAMOSA-promoter binding proteins (SBP). It is likely that miR529 is either related to, or is a sub-grouping of, the miR156 family, but with a distinct tissue expression profile.

As seen in the miR396 gene family, tissue expression profiles are not necessarily conserved amongst miRNA genes of the same family. This family consists of 7 members that exhibit distinct expression profiles (Figure 4, Table S2) – miR396a/b/g shows elevated expression in the pollen, miR396c/d is highly expressed in the juvenile tissues but not adult tissues, while miR396e/f expression profile shows a peak in seedling tissues. miR396c/d mature miRNA sequences carry an additional guanine residue between positions 8 and 9 as compared to the rest of the miR396 family. Other members of the miR396 family are conserved in both monocots and dicots, but miR396c/d appear to be monocot specific (equivalent to osa-miR396d/e found in rice) [63]. The latter are also the only members of the miR396 family that target QLQ (glutamine-leucine-glutamine) and WRC (tryptophan-arginine-cysteine) domains that define the Growth-Regulating Factor (GRF) family of transcription factors [64]. The GRF transcription factors are involved in leaf and cotyledon growth and expressed most abundantly in active developing tissues [65]. QLQ has been found to be involved in mediating protein interactions whereas WRC plays a role in DNA binding [64]. Therefore, miR396c/d could be acting as regulatorsr of GRF genes in juvenile tissues independently from the rest of the miR396 family.

There are also miRNA gene families that appear to be constitutively expressed in all 5 tissues. For example, miR168 family has a read count of at least 2400 RPM in all tissues surveyed, reflecting its role in maintaining a steady-state balance of the RNA silencing machinery by targeting the slicer AGO1 (ARGONAUTE1) of the RISC complex [66]–[69].

Recent transcriptome analysis of Arabidopsis indicates that 15 genes involved in the miRNA pathway (including AGO1, AGO2, AGO4 AGO7, and DCL1–3) are absent in pollen [70]. In the current study, we observed that expression of most families are lower in pollen as compared with other tissues, suggesting that the miRNA biogenesis machinery is similarly down-regulated in maize pollen tissues.

Of the 26 miRNA families on our refined list, only miR482 (consisting of a single member) failed to register any evidence of expression in the 5 tissues sampled. While miR482 had previously been annotated in poplar, pine, soybean and grape, it has not been identified in any monocots.

### Characterization of pri–miRNA transcripts

Our analysis of the pri-miRNA transcripts shows that these genes exhibit many of the conserved signatures of RNA polymerase II transcripts. These include TATA boxes in conserved locations, alternative TSS and polyadenylation sites, and introns [39]. Using the stems of the pre-miRNA for orientation, we found that, on average, the 5′ region is shorter than the 3′ region. The average length of the 3′ region is likely an underestimation due to ascertainment biases inherent in the RACE protocol, where smaller transcripts are more likely to be amplified than longer ones. This may also explain the shorter average length of pri-miRNAs amplified by RACE as compared with the average length of FL-cDNA that harbor pre-miRNA sequences (Table S3). The frequency of TATA-box appearance (83%) is similar to that of Arabidopsis miRNA transcripts (83%) [39] and much higher than average for protein promoters (50%) [43]. As in [39], we also found a high occurrence of cytosine at the −1 position and adenine at the +1 position of TSSs (Figure 2). Together, such characteristics as the TATA box, the nucleotide frequency surrounding the TSSs and expected size of 5′ transcript regions, will help in the development of new methods to predict proximal promoters of miRNA genes.

### Target prediction

Our target prediction method is stringent, but still allows us to capture most miRNA targets that are conserved across several plant species, including Arabidopsis [4], [71]–[73], poplar [21], rice [67],[74],[75], wheat [76], soybean [77], mustard [78], and grape [79]. For example, miR156 targets SBP transcription factors [80],[81], while miR159 targets the MYB family [82]. Both miR160 and miR167 target Auxin Response Factor (ARF) transcription factors in Arabidopsis and maize [83],[84]; and are also captured by our predictions. The same trend is observed in many other miRNA families including miR164, miR166, miR169, miR171, miR172, miR319 and miR396 as they target various families of transcription factors such as NAM (No Apical Meristem) proteins, bZIP (basic-leucine Zipper) genes, CBF (CCAAT binding factor), GRAS transcription factor, AP2 (APETALA2)-EREBP (Ethylene-Responsive Element Binding Proteins), CCCH type zinc finger protein and TCP (Teosinite branched, Cycloidea, and PCF), GRF transcription factor families respectively [12], [71], [85]–[87]. These transcription factors are known to regulate plant development. MiRNA families miR393 and miR394 target F-box protein and are known to play a role in the expression control of genes involved in regulation of metabolic processes [88]. It has been reported that in Arabidopsis, rice, and wheat, miR168 targets AGO1 (ARGONAUTE1) [66]–[69]. The prediction pipeline captured three AGO1 orthologs in maize [89]. Our target predictions are further consistent with the literature in the case of miR395, which regulates sulfate metabolism by targeting sulphate transporter genes and ATP sulfurylase (APS) proteins, and whose expression increases in response to low sulfate growing conditions [73].

We also predicted potential targets of miRNA families not previously identified in maize (miR482, miR528, miR529, miR827 and miR1432). Trehalose phosphatase, cytochrome P450, pentatricopeptide, and CCHC type zinc finger protein were predicted as targets of miR482, suggesting the involvement of miR482 in a wide range of biosynthetic reactions. Similar to miR397 and miR408, miR528 also targets copper proteins cupredoxin, multicopper oxidase and laccase genes and thus might play a critical role in regulating physiological processes (photosynthetic and respiratory electron transport) and stress responses. Both miR156 and miR529 were predicted to target genes encoding the SBP box. miR827 was predicted to target NAD(P)-binding and SPX (SYG1/Pho81/XPR1) proteins, whereas miR1432 was predicted to target Poly(ADP-ribose) polymerase, catalytic region functions and calcium binding EF hand domains. Both SPX and calcium binding EF hand domains are associated with proteins that are active in signal transduction pathways.

The stringent criteria used to predict targets could potentially reduce false positive rates at the cost of missing several authentic targets. For example, miR162 targets DCL1 [90], but this target (GRMZM2G040762 in maize) was excluded by our pipeline due to the presence of a 1nt bulge in the alignment with miR162. In addition, for about 15% of the predicted targets, functional information is not available at this time. We hypothesize that some of these targets may be novel but this needs to be further verified based on experimental evidence.

### Retention of miRNA genes following whole-genome duplication

The maize lineage tetraploidy event occurred an estimated 5 to 15 MYA [91]–[94], and precipitated large-scale chromosomal rearrangements as well as massive losses of duplicate genes in the process of returning to a genetically diploid state [26], [94]–[97]. Our analysis showed that greater than 1/3 of ancestral miRNA positions were retained at both homoeologous sites and these accounted for the majority of gene family expansions in maize relative to sorghum. Interestingly this proportion of retained sites was greater than that seen for protein-coding genes. Gene duplication, especially by polyploidization, has long been thought to provide raw material for the evolution of functional novelty [98]. The initial redundancy of duplicate genes is followed by a period of relaxed selection with gene loss being the ultimate fate for most duplicates [99],[100]. Classical models for retention of duplicate homoeologs include neofunctionialization, subfunctionalization, and maintenance of stochiometry among interacting components of pathways [101]. Evidence for subfunctionalization has been reported in tandem duplicate paralogs of miR168 in the Brassicaceae[50]. However, more recent hypotheses on the causes of retention following genome-wide duplication events are based on common characteristics of retained genes. For example, studies of the after effects of polyploidy in Arabidopsis [102], rice [103] and most recently maize [26] show bias for retention of transcription and other regulatory factors. Such classes may have indispensable functions such that their retention provides buffering against gene loss [104]. As shown here and elsewhere, miRNA genes are largely associated with regulatory functions and thus mutations in miRNAs can be expected to have profound deleterious effects on growth and development [62]. Thus the observed preferential retention of miRNA genes compared to protein coding genes is consistent with both the genetic buffering hypothesis and with previous observations of biased retention of regulatory genes.

The majority of maize miRNA genes (91%) lie within ancestral locations that existed prior to the speciation of maize and sorghum. Those no longer at syntenic positions could represent lineage-specific movements, duplications, or losses. Identification of ortholog genes has important practical application, allowing inferences to be drawn between functional studies in each species. Conservation of gene position has proven useful for determining orthologous relationships, particularly in cases where multiple highly related paralogs exist at different genome locations [105],[106]. Such is the case for miRNA genes, which tend to occur in large yet highly conserved families. Although synteny provides a strong indication of orthology, potentially complex relationships are suggested in the case of paralog clusters, in which closer phylogenetic analysis may be required to sort out duplication histories [50]. Nevertheless, we found simple synteny relationships for 69 sorghum genes and 81 maize genes, allowing presumptive assignment of orthology among these genes.

### Conservation of mature miRNA in diverse lines

To investigate the evolution of miRNA loci we sequenced 28 loci and flanking regions in panels of inbred and teosinte lines. This germplasm set was selected as it provides an excellent representation of the allelic diversity in maize and wild relatives. As expected, there was no polymorphism detected within the mature miRNA sequences. Their conservation within maize throughout its evolution is expected given the importance of miRNA genes in suppressing target gene expression during development and stress. The flanking regions displayed diversity levels similar to protein coding genes indicating that purifying selection is limited to mature miRNAs. None of the 28 loci tested exhibited the extreme reductions in diversity in inbreds relative to teosinte accessions that would be indicative of artificial selection during domestication or crop improvement. MicroRNA loci may control such fundamental processes in development that alterations of sequence or expression are not tolerated. Our results are similar to studies in Arabidopsis [107],[108] and rice [35] where strong purifying selection has acted on the mature miRNA, whereas flanking regions generally reflect species wide diversity levels.

In summary, we have investigated genome-wide maize miRNA genes from several aspects: their pre- and pri- structure, their expression level, their targets, their conservation, and their evolution, providing evidence of the important function of miRNAs in regulating metabolic and developmental process and adaptation to stress. Identification and characterization of this important class of regulatory genes in maize may enable breeders to engineer crops with improved architecture and stress responses necessary to increase yields for food and fuel, while addressing environmental concerns in our changing climate and ever-increasing human population.

## Materials and Methods

### Plant materials

Maize (*Zea mays*) inbred line B73 was used in this study. The seedlings were collected from plants grown in soil in a green house at 22°C with a 16 hr light cycle and harvested 7 days after germination. Roots were collected from B73 seeds grown in sterile water without light at 30°C for three days. Immature ear (size 0.5–2cm) and immature tassel (size 0.5–2.5cm) were harvested from plants grown in the field. All the tissues were harvested and immediately frozen in liquid nitrogen and stored at −80°C. Tissues used in the RACE analysis included seedlings and immature inflorescence (tassel and ear).

### Pre–miRNA predictions

A list of plant mature miRNAs were obtained from miRBase (version 13.0) and aligned using Vmatch, an alignment algorithm that employs suffix arrays (www.vmatch.de), against a suffix array index of the maize genome sequence built using mkvtree from the vmatch package. Up to 2 mismatches were allowed in the alignment. A 250-nt contextual sequence surrounding each aligned mature miRNA was extracted and secondary structure modeling of the DNA fragment was performed using Mfold [109]. Segments that could potentially form the canonical stem-loop signature of a precursor miRNA were tagged as putative miRNAs. This putative list was further trimmed based on the following criteria: (1) overlapping loci were reduced to non-redundant representations, (2) loci that overlapped transposable and other repetitive elements were removed, (3) loci in which the matched mature miRNA was in the wrong orientation were removed, (4) each predicted secondary structure was curated manually and those with spurious stem-loop structures were removed [73], and (5) miRNA families that had no evidence of expression in the small RNA libraries or RACE were removed (for more details see Figure S2). The final refined list of predicted miRNAs consisted of 150 individual miRNAs from 26 miRNA families. Based on the hairpin structure of the pre-miRNA, the mature miRNA and corresponding miRNA* sequence were identified for each.

### Small RNA libraries for sequence-by-synthesis (Illumina):

We used a modified method based on Llave *et al* to prepare small RNA libraries [2]. Total RNA from different tissues was extracted with TRIzol solution (Invitrogen). 100µg of total RNA were separated on 15% Acrylamide/8M Urea gel (Sequagel, National Diagnostic) along with 19 and 24nt RNA samples as marker. Small RNAs were extracted from the gel slices corresponding to 19 and 24nt RNA using high salt. Small RNAs were ligated with Modban (Linker 1 from IDT). Ligated samples were separated again on 15% Acrylamide/8M Urea gel. The gel fragments corresponding to 37–42nt were excised. Small RNA was purified from the gel fragments, and 5′ sequencing adaptors were added using T4 RNA ligase. This RNA was amplified using Superscript III Reverse transcriptase (Invitrogen) and PCR amplification (Phusion HF polymerase, NEB). Amplified cDNA were separated on 3% MetaPhor Agarose (VWR) and the bands corresponding to correct size (108–115nt) were cut and purified with QIAGENE gel purification kit and sent for Illumina sequencing.

### Small RNA sequencing and sequence processing

Small RNA libraries were sequenced on an Illumina Genome Analyzer using the 36-cycle Solexa Sequencing Kit (Illumina). The Illumina Gerald pipeline was used to process and extract the first 36 bases of the runs. Adaptor sequences were identified and trimmed from each read using a customized Perl script. Reads in which the adaptor could not be identified were discarded. Novoalign (version 2.03, http://www.novocraft.com/) was used to align the trimmed reads to the high-confidence set of 150 pre-miRNAs. For each library, we counted the number of trimmed reads within the 18–22nt range that were mapping to each pre-miRNA and normalized by the total number of 18–22nt trimmed reads in the library. Trimmed reads that were <18nt or > = 23nt were not considered in this analysis.

### RACE analysis of miRNA precursor

Total RNA was extracted from maize seedlings and immature inflorescence using Spectrum Plant Total RNA kit (Sigma) and treated with RNase Free DNase (QIAGEN). cDNA templates were prepared by following the instruction of FirstChoice RLM-RACE Kit (Ambion). For each miRNA precursor, two sets of gene-specific primers, mostly immediate upstream and downstream of predicted hairpin structures (for 5′ RACE, for 3′ RACE, respectively), were designed using Primer 3 [110](Table S5). These primers were used for two rounds of PCR amplification. Nested PCR products were analyzed on agarose gel. Positive PCR products were cloned into pCR2.1-TOPO vector using TOPO TA cloning kit (Invitrogen). Each transcript sequence was confirmed by at least 8 unique clones. Sequences corresponding to the transcripts were mapped to the genome and listed in Table S3.

### Computational prediction of miRNA protein-coding target transcripts

The miRNA target transcript prediction pipeline was developed using Vmatch. For the maize protein coding transcripts, the predicted cDNA of the longest consensus maize transcript from the filtered gene sets were used in this analysis and indexed by using mkvtree (Vmatch)[26]. The mature miRNA sequences from the refined miRNA set were reverse complemented and matched against the indexed maize transcript database, with the parameters relaxed to allow up to six mismatches. The matched miRNA target transcripts were further filtered by applying empirical rules defined by Schwab *et al* [111]. Briefly, we scored each miRNA complementary site. Perfect matches were given a score of 0, and all other mismatches were scored 1. Only 1 mismatch score was allowed between positions 2 to 12 inclusive. However, no mismatches were allowed at position 10 and 11 and no more than 2 consecutive mismatches were allowed after position 12. A maximum of three mismatches (excluding mismatch at position 1) was allowed across the length of the mature miRNA.

### Maize miRNA targets functional enrichment analysis

We subjected potential miRNA targets for functional enrichment analysis against GO molecular function and GO biological process terms database by using BiNGO [48] which is a Cytoscape [49] plugin that maps over-represented functional themes present in a given gene-set onto the GO hierarchy. For enrichment P-value calculation (at a significance level of 0.05 or better), a hypergeometric distribution statistical testing method was selected to ensure that target genes are not hitting their corresponding biological function/process classes purely by random chance. For multiple hypotheses testing, the Benjamini and Hochberg false discovery rate (FDR) correction [112] was applied to reduce false negatives at the cost of a few more false positives.

In order to fully understand the function of each target, we also extracted information based on phylogenetic trees of putative genes from maize, sorghum, Arabidopsis, and rice genomes. The trees were computed using the Ensembl Compara GeneTree method [113]. The input sequences used for the GeneTree analysis were the longest translation at a given gene locus, filtered for transposons and other low-confidence genes, from the following genome annotation resources: the maize genome (www.maizesequence.org) release 4a from June 2009 [26], the Sorghum genome [29] JGI release Sbi 1.4 from March 2008, The Arabidopsis Information Resource [114] release 8 from April 2008, and the MSU/TIGR Rice Genome Annotation Resource [115] release 5 from January 2007.

### Synteny analysis of maize and sorghum

Shared synteny among homologous maize and sorghum miRNA genes was evaluated in the context of orthologous protein-coding genes. Orthologous protein-coding genes were identified using the Ensembl Compara pipeline, which is based on a phylogenetic analysis [116]. In all, the orthologue sets included 25,216 maize genes and 20,408 sorghum genes, which participated in 27,275 relationships. DAGchainer [117] was used to identify colinear chains amongst these orthologs, together with all pairwise combinations of within-family miRNA genes. Such chains were required to have at least five colinear genes with no more than ten intervening genes between neighbors. Additional syntenies among non-colinear genes were searched based on distance to colinear anchors that flank the orthologue of the gene in question in the other genome. The gene in question was considered syntenic if positioned within five genes of its nearest anchor. Paralogous clusters of miRNA genes were identified as family members separated by no more than two intervening genes.

### Diversity of miRNA loci

Two diverse sets of maize materials were used for DNA sequence analysis: maize inbred lines, and partially inbred teosinte accessions (Table S11). The 28 maize inbred lines (B73, B97, CML103, CML228, CML247, CML277, CML322, CML333, CML52, CML69, Hp301, IL14H, Ki11, Ki3, Ky21, M162W, M37W, Mo17, Mo18W, MS71, NC350, NC358, Oh43, Oh7B, P39, Tx303, Tzi8 and W22) were chosen to maximize allelic diversity. The 16 partial inbred teosinte (*Zea mays ssp. parviglumis*) accessions are identical to those used previously [56],[57]. We conducted extended DNA sequencing of the known 28 maize miRNAs. Primer 3 was used to design the sets of PCR primers for analysis of the selected candidate miRNA genes. The genomic PCRs were performed using PCR master Mix I (Promega) or Takara LA Taq polymerase. Unincorporated primers and dNTPs were removed by exonuclease I (NEB) and shrimp alkaline phosphatase (USB). The PCR products were ethanol precipitated and sequenced with forward, reverse, and internal primers using Illumina machines. Base calling, quality assessment, and trimming of trace files were conducted with PHRED and sequence assembly was performed by PHRAP. The multiple sequences for each gene were aligned with ClustalW. We used DNAAlign Editor [118] for aligning the amplicon sequences across the germplasm set to accurately identify nucleotide variations, insertions, and deletions. The alignments are available to the community from the www.panzea.org [119].

The data discussed in this publication have been deposited in NCBI's Gene Expression Omnibus [120] and are accessible through GEO Series accession number GSE17943 (http://www.ncbi.nlm.nih.gov/geo/query/acc.cgi?acc=GSE17943).

## Supporting Information

Table S1 miRNA families removed based on TE annotation.
(0.04 MB DOC)

Table S2 High-confidence maize miRNA genes.
(0.06 MB XLS)

Table S3 Maize miRNA genes mapped to maize genome.
(0.04 MB XLS)

Table S4 Maize miRNA genes with intron.
(0.04 MB DOC)

Table S5 Primers used in 5′ and 3′ RACE.
(0.03 MB XLS)

Table S6 Complete list of predicted miRNA targets of maize miRNAs. For each miRNA family, total number of targets, their function, Arabidopsis orthologs, and Interpro IDs are given.
(0.10 MB XLS)

Table S7 Functional classification of targets with miRNA families based on Gene Ontology (GO). Classification of miRNA families of significantly overrepresented predicted miRNA targets based on GO full annotations are given. Adjusted P value, based on Benjamini and Hochberg correction applied on hypergeometric test is given. Cluster frequency represents total number of genes annotated to that GO term divided by total no of genes in the test set. Total frequency represents total number of genes annotated to that GO term divided by total no of genes in the reference set. Since one gene can be related to multiple GO terms, the sum of the percentages in each column is not a relevant parameter.
(0.05 MB XLS)

Table S8 miRNA genes in maize and sorghum.
(0.02 MB XLS)

Table S9 Syntenic sites harboring MIR genes in maize and sorghum. The 82 ancestral sites are numbered according to their order in sorghum. “Homoeologue status” indicates whether the site is retained on only one or both homoeologous positions in maize. If “Single” then information is provided under “Maize Homoeologue A.” If “Both” then information is additionally given under “Maize Homoeologue B.” “Size” indicates the number of clustered paralog genes at a site. Only one representative gene is listed for each site; for paralog clusters this is the first in the series.
(0.04 MB XLS)

Table S10 Panzea accession of maize miRNA genes.
(0.04 MB XLS)

Table S11 Maize miRNA gene diversity.
(0.03 MB XLS)
