# Supplementary material for: A Genome-Wide Characterization of MicroRNA Genes in Maize
Source: PLoS Genet. 2009 Nov 20;5(11):e1000716. doi: 10.1371/journal.pgen.1000716 (PMC2773440; doi:10.1371/journal.pgen.1000716)
Supplement: Table S1 — miRNA families removed based on TE annotation. (0.04 MB DOC) [file pgen.1000716.s001.doc]

| Table S1: miRNA families removed based on TE annotation | | | |
| --- | --- | --- | --- |
| miRNA Family | Total Numbers | Numbers located in TE | TE percentage |
| miR437 | 94 | 90 | 96% |
| miR854 | 1 | 1 | 100% |
| miR1128 | 14 | 14 | 100% |
| miR1132 | 4 | 4 | 100% |
| miR1133 | 7 | 4 | 57% |
| miR1320 | 1 | 1 | 100% |
| miR1435 | 14 | 8 | 57% |
| miR1436 | 21 | 21 | 100% |
| miR1439 | 43 | 41 | 95% |
| miR1884 | 1 | 1 | 100% |
| miR2102 | 2 | 1 | 50% |
| Total | 202 | 186 |  |

TE: Transposable Elements
