# Supplementary material for: A Genome-Wide Characterization of MicroRNA Genes in Maize
Source: PLoS Genet. 2009 Nov 20;5(11):e1000716. doi: 10.1371/journal.pgen.1000716 (PMC2773440; doi:10.1371/journal.pgen.1000716)
Supplement: Table S4 — Maize miRNA genes with intron. (0.04 MB DOC) [file pgen.1000716.s004.doc]

| Table S4: Maize miRNA genes with intron | | | |
| --- | --- | --- | --- |
| MIR Gene | Orientation to HP^a^ | Intron size (nt) | Spliced Sites |
| MIR156c/b | 3' | 94 | GU…AG |
| MIR156e | 3' | 288 | GC…UG |
| MIR159a | 3' | 91 | GU…AG |
| MIR164c | 5' | 71 | GU…AG |
| MIR166f | 3' | 72 | GU…AG |
| MIR166c | 3' | 1787 | GU…AG |
| MIR166g | 3' | 2196 | GU…AG |
| MIR167d | 3' | 271 | GU…AG |
| MIR167e | 3' | 262 | GU…AG |
| MIR167i | 3' | 137 | GU…AG |
| MIR169h | 3' | 522 | GU…AG |
| MIR169i | 3' | 108 | GU…AG |
| MIR169k | 3' | 404 | GU…AG |
| MIR171i | 3' | 615 | GU…AG |
| MIR393a | 3' | 294 | GU…AG |
| MIR399c^b^ | 3' | 329 | GU…AG |
| MIR399c^c^ | 3' | 856 | GU…AG |

^a^HP: hairpin

^b^first intron

^c^second intron
